# A newborn with a necrotic scalp lesion

**DOI:** 10.1016/j.jdcr.2025.03.010

**Published:** 2025-03-28

**Authors:** Caroline Kruithoff, Diana Mustafa, Ahmed Sorour

**Affiliations:** a Ohio University Heritage College of Osteopathic Medicine, Cleveland, Ohio; b Department of Plastic Surgery, Mataria Teaching Hospital, Cairo, Cairo Governorate, Egypt

**Keywords:** anterior fontanelle, aplasia cutis congenita, necrotic, scalp lesion, skull defect

## History

**Fig 1:**
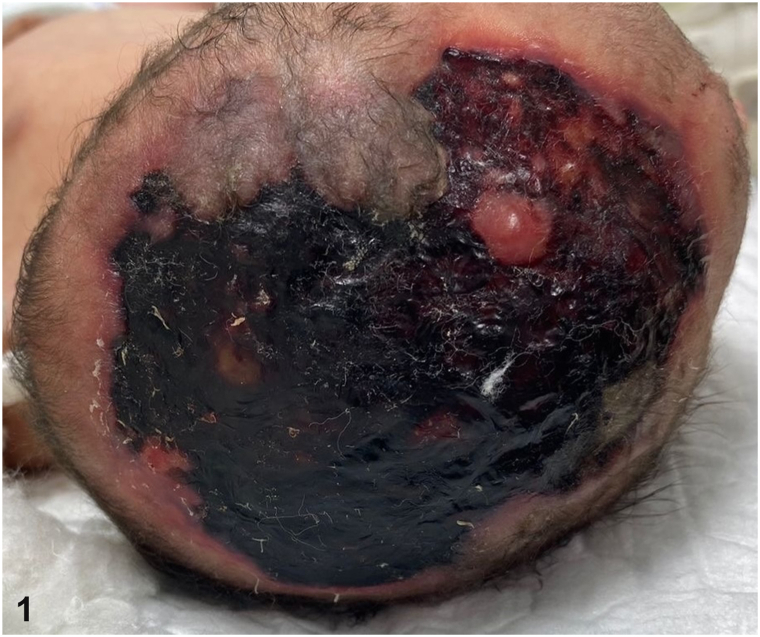

**Fig 2:**
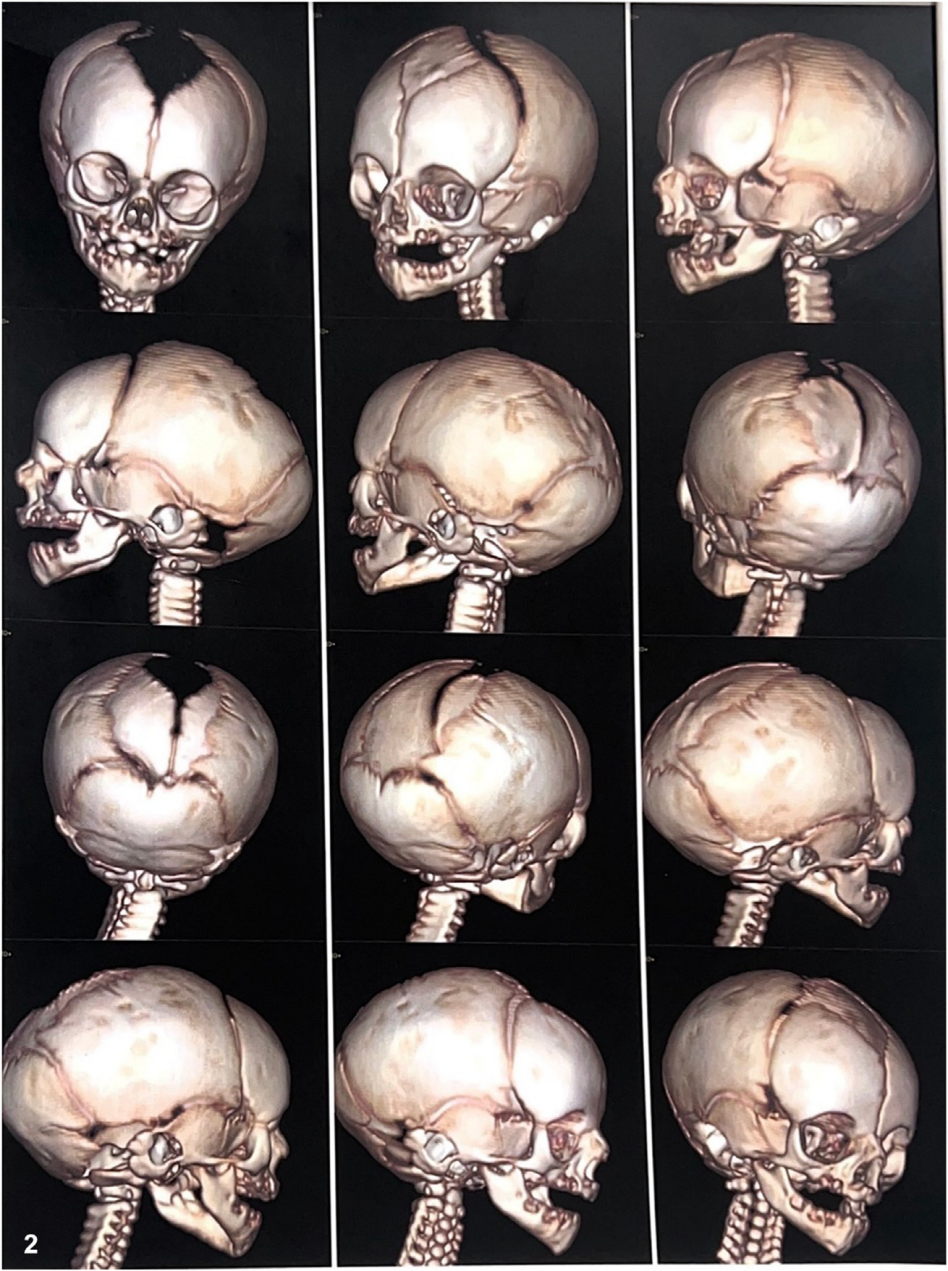

A male newborn was delivered at full term via cesarean section to a 35-year-old G5P4 female with little prenatal care. She was diagnosed with pre-eclampsia in the first trimester and treated with nifedipine and aspirin. During pregnancy, she also received cefotaxime, lactoferrin, and tiemonium methylsulfate. There were no known prenatal viral exposures or birth trauma. The newborn presented with a 12 cm × 6 cm necrotic scalp lesion (Fig 1). A computed tomography scan of the head with 3D reconstruction confirmed a widened anterior fontanelle (Fig 2). Of note, the mother had an asymptomatic scalp lesion that had been present since birth.

**Question 1: Which of the following is the most likely diagnosis?**

A. Aplasia cutis congenita
B. Congenital triangular alopecia
C. Morphea en coup de sabre
D. Nevus sebaceus
E. Congenital alopecia areata

**Answers:**

A. Aplasia cutis congenita – Correct. Aplasia cutis congenita (ACC) is a rare congenital condition characterized by focal or extensive absence of the epidermis, dermis, and subcutaneous tissue and may be associated with underlying skull defects, such as a widened anterior fontanelle. While the diagnosis is made clinically, histopathology shows deep reaching scars and fragmented elastic fibers, which differentiates ACC from other forms of congenital alopecias.^1^
B. Congenital triangular alopecia – Incorrect. Congenital triangular alopecia most commonly affects the frontotemporal scalp and presents as a triangular patch of hair loss. Histopathology shows sparse vellus hair follicles that have replaced terminal hair follicles.^1^
C. Morphea en coup de sabre – Incorrect. Morphea en coup de sabre is a type of linear localized scleroderma that presents as a linear indurated plaque on the frontoparietal scalp. Histopathology shows classic features of scleroderma and preserved elastic fibers.^1^
D. Nevus sebaceus – Incorrect. Nevus sebaceus is a congenital lesion characterized by a hairless, yellow-orange plaque, most often on the scalp or face. Histopathology shows epidermal changes such as acanthosis and papillomatosis.^1^
E. Congenital alopecia areata – Incorrect. Congenital alopecia areata presents as patches of nonscarring hair loss that can affect the entire scalp. Histopathology shows small anagen follicles with lymphocytic infiltration.^1^

**Question 2: Which of the following anatomical locations does ACC most commonly affect?**

A. Face
B. Abdomen
C. Lower extremity
D. Upper extremity
E. Scalp

**Answers:**

A. Face – Incorrect. ACC may present as linear facial defects in the setting of various rare genetic syndromes. However, this is an uncommon presentation.^2^
B. Abdomen – Incorrect. While ACC has been reported to occur on the abdomen, this presentation is uncommon. Abdominal ACC may be associated with fetus papyraceus.^3^
C. Lower extremity – Incorrect. This presentation is uncommon and may be seen in Adams-Oliver Syndrome or epidermolysis bullosa with congenital absence of skin (previously known as Bart Syndrome), both of which are rare genetic disorders.^4^
D. Upper extremity – Incorrect. This presentation is uncommon and may be associated with Adams-Oliver Syndrome, a condition characterized by transverse limb defects. However, the lower extremities are more frequently involved.^4^
E. Scalp – Correct. ACC most commonly occurs on the scalp vertex. Although ACC can occur as an absence of skin at other anatomical sites, these presentations are much less common.^1^

**Question 3: All of the following have been associated with ACC, except** **which of** **the following:**

A. Adams-Oliver Syndrome
B. Forceps delivery
C. Heterozygous missense mutation in BMS1
D. Knobloch Syndrome
E. SCALP Syndrome

**Answers:**

A. Adams-Oliver Syndrome – Incorrect. This syndrome primarily presents with ACC on the vertex of the scalp along with transverse terminal limb defects, cardiac malformations, and central nervous system abnormalities.^4^
B. Forceps delivery – Correct. Lesions resulting from forceps delivery or other birth trauma have been mistakenly identified as ACC in the past and must be excluded. ACC is a sporadic intrauterine occurrence that is not associated with these events.^5^
C. Heterozygous missense mutation in BMS1 – Incorrect. This mutation, which affects ribosomal function, has been identified in the autosomal dominant variant of ACC and may explain the possibility of a familial inheritance in this patient’s case, given the mother’s scalp lesion.^4^
D. Knobloch Syndrome – Incorrect. This syndrome can present with ACC affecting the skull, as well as occipital abnormalities affecting both the skull and eyes, including retinal detachment and occipital primary encephalocele.^4^
E. SCALP Syndrome – Incorrect. This syndrome is characterized by symmetrical ACC on the scalp, often accompanied by epidermal nevi. Other features of SCALP syndrome include sebaceus nevi, central nervous system malformations (such as seizures, developmental delays, and brain tumors), limbal dermoids, and pigmented nevi.^4^

## Conflicts of interest

None disclosed.
